# Inflammation and Oxidative Damage in Fatty Acids Oxidation Disorders

**DOI:** 10.1002/cbf.70328

**Published:** 2026-11-03

**Authors:** Stefani Altenhofen, Luísa Tedesco, Franciele Fátima Lopes, Marina Acosta, Ângela Sitta, Daniella de Moura Coelho, Bianca Gomes dos Reis, Luiza Wailer, Debora Tonelotto, Ana Kalise Bottcher, Moacir Wajner, Carmen Regla Vargas

**Affiliations:** ^1^ Universidade Federal do Rio Grande do Sul (UFRGS) Porto Alegre Rio Grande Brazil; ^2^ Hospital de Clínicas de Porto Alegre (HCPA) Porto Alegre Rio Grande Brazil

**Keywords:** carnitine, fatty acid oxidation disorders, inborn errors of metabolism, inflammation, oxidative stress

## Abstract

Deficiencies in enzymes and transporters involved in mitochondrial fatty acid beta oxidation results in the accumulation of fatty acids, a condition known as fatty acid oxidation disorders (FAODs). Therefore, the present study aimed to evaluate the oxidative and inflammatory profiles of treated patients with FAODs, comparing those receiving dietary treatment and l‐carnitine supplementation with one untreated FAOD patient, to provide new insights into the pathophysiology and treatment of this group of diseases. Samples from 12 treated FAOD patients, 18 age‐matched controls, and one untreated patient at diagnosis were included in the study. Levels of 8‐isoprostanes, DNA and RNA oxidative damage, nitrite and nitrate, cathepsin D and cytokines were measured in urine and/or plasma samples, depending on the analyte, of FAODs patients under treatment (mean: 3.0 ± 2.9 years) and of an untreated patient with increased levels of several organic acids, who was included as an illustrative case and not considered in the statistical analyses. Levels of 8‐isoprostanes, nitrite and nitrate, DNA damage, and interleukins 1β, 8, and 10 did not differ significantly between treated patients and control group, whereas IL‐6 levels were increased in FAODs patients. These findings indicate that treated FAOD patients presented oxidative and inflammatory biomarker levels similar to those of controls for most analytes, while IL‐6 remained elevated. The results suggest a potential beneficial effect of current treatment on oxidative and inflammatory profiles.

## Significance Statement

Mitochondrial fatty acid beta‐oxidation disorders are a group of rare enzyme deficiencies with pathophysiological mechanisms that have not been completely elucidated yet, although it is known that there is the involvement of processes such as mitochondrial dysfunction, inflammation and oxidative stress. The current work is the first to evaluate the impact of treatment upon oxidative and inflammatory parameters in patients with FAODs.

## 1 Introduction

Acyl‐CoA dehydrogenases are a group of enzymes involved in the mitochondrial fatty acid β‐oxidation pathway, which promotes the catabolism of fatty acids to maintain cellular homeostasis and energy metabolism through the generation of Acetyl‐CoA, FADH_2_, and NADH, supplying the TCA cycle and oxidative phosphorylation [1, 2]. The dehydrogenases are classified according to the respective fatty acids used as substrates, including medium‐chain (MCAD), long‐chain hydroxy (LCHAD), and very long‐chain (VLCAD) dehydrogenases. Autosomal recessive mutations may result in deficiencies of these enzymes (LCHADD, VLCHADD, MCADD, respectively), leading to a group of inborn errors of metabolism known as fatty acid β‐oxidation disorders (FAODs) [3, 4]. This group also includes multiple acyl‐CoA dehydrogenase, also known as glutaric aciduria type II, which is caused by alterations in genes encoding electron transfer flavoprotein (ETF) and its dehydrogenase (ETFDH). This defects impair the transfer of electrons from acyl‐CoA dehydrogenases do ubiquinone, an important step in oxidative phosphorylation [5]. Globally, FAODs present an overall prevalence of approximately 1 in 15,000 lives births, with MCADD being most common disorder [5, 6].

Clinically, both the age of onset of clinical manifestations and the severity of symptoms in FAODs can vary widely among pacients [4]. Affected individuals present hypoketotic hypoglycemia, resulting from impaired gluconeogenesis and ketogenesis, as well as disfunction of organs that rely heavily on lipid metabolism, leading to manifestations, such as encephalopathy, arrhythmias, and hepatopathy [7, 8]. The complete elucidation of the pathophysiology of these conditions remains under investigation. However, it is well established that the accumulation of fatty acids and their metabolic derivatives lead to toxicity, contributing at least partially to the aforementioned symptoms [8]. Conjugation of fatty acids with l‐carnitine (l‐car) results in a characteristic acylcarnitines profile, which is considered the primary method for FAODs screening, followed by confirmation obtained through molecular analysis or enzymatic assays performed in fibroblasts, lymphocytes or tissues samples [9].

Current treatment for FAODs include the l‐carnitine supplementation (50–100 mg/kg/day)—which is often decreased in patients—as well as dietary management consisting of avoidance of long periods of fasting, restriction in fats, and increased carbohydrates intake. VLCADD patients constitute an exception, as l‐car supplementation may increase the accumulation of long‐chain acylcarnitines [10], although clinical management may vary according to medical evaluation. These patients may require supplementation with medium‐chain triglyceride oil and docosahexaenoic acid [11]. Other potential therapeutic options for FAODs include triheptanoin and bezafibrate; however, further studies are required to adequately establish their clinical benefits [11]. Although current treatments reduce hospitalization rates and improve clinical outcomes, dietary management alone is considered insufficient to prevent certain complications, especially rhabdomyolysis and cardiomyopathy, which may result in fatal outcomes [12, 13].

Different pathologies are known to cause mitochondrial dysfunction (MD), which is associated with impaired antioxidant defenses, increased generation of oxygen reactive species (ROS), and the release of inflammatory mediators [8]. Previous studies already been suggested the occurrence of ROS formation and mitochondrial damage in patients with FAODs, with a significant increase in oxidative stress biomarkers in patients and animal models during metabolic decompensation [8, 14, 15, 16, 17, 18]. However, evidence regarding the impact of treatment on these parameters remains limited.

Therefore, the present study aimed to evaluate the oxidative and inflammatory profiles of treated patients with FAODs, comparing those receiving dietary treatment and l‐carnitine supplementation with one untreated FAOD patient, to provide new insights into the pathophysiology and treatment of this group of diseases.

## 2 Materials and Methods

### 2.1 Patients and Biological Samples

Plasma and urine samples were obtained from 12 FAODs patients under treatment and 1 FAOD patient at diagnosis moment from the REDE‐EIM Brasil of Medical Genetic Service of Clinical Hospital of Porto Alegre (LCHADD at treatment, *n* = 3; LCHADD at diagnosis (illustrative case), *n* = 1; MCADD, *n* = 1; MADD, *n* = 5; VLCADD, *n* = 3). Due to the rarity of FAODs, all patients with a confirmed diagnosis and available biological samples were included in the study.

Samples were obtained by standard clinical procedures, with plasma separated by centrifugation. Samples were stored at −80°C until analysis. Dried blood spots (DBSs) were used for acylcarnitine analysis. All biomarkers were measured in their respective matrices (plasma, urine or DBSs, as specified in each assay), and no normalization procedures were applied.

Due to the small sample size inherent to the rarity of each FAOD subtype, subgroup analyses were not feasible, and patients were analyzed as a combined FAOD group to allow exploratory evaluation of oxidative and inflammatory biomarkers. Differences in sample size among biomarkers are due to limited sample volume available for all analyses and, in some cases, exclusion of samples based on quality control criteria and/or statistical outlier identification.

The mean age of the patients was 14 ± 13.51 years. The average treatment time was 3.0 ± 2.9 years. Samples from 18 individuals with matching ages were used for the control group. A sample of a LCHADD 1‐year‐old patient at the moment of diagnosis (presenting hepatomegaly, hypoglycemia, hypotony, convulsions and delay in neuropsychomotor development and growth) was obtained and included as an illustrative case. This patient was not considered in the statistical analyses. All treated patients, including those with VLCADD deficiency, were under dietary management and received l‐carnitine supplementation (100 mg/kg/day). The diagnosis of all patients was previously confirmed by the analysis of organic acids by gas chromatography coupled to mass spectrometry using urine samples. This study was approved by the Ethics Committee of Clinical Hospital of Porto Alegre (21‐0424 – Clinical Trial Number: not applicable).

### 2.2 Determination of Organic Acids

Organic acids were determined in urine samples through gas chromatography coupled to mass spectrometry (GC/MS), using hexadecane and heptadecanoic acid as internal standards for quantification [19].

### 2.3 Determination of Acylcarnitines

Acylcarnitines were determined in DBSs using liquid chromatography coupled to tandem mass spectrometry (LC‐MS), using the multiple reaction monitoring mode (MRM) [20].

### 2.4 Determination of DNA/RNA Oxidative Damage

Oxidative damage to DNA and RNA was assessed in plasma samples using the commercial kit DNA/RNA Oxidative Damage Elisa (Cayman Chemical, item code 589320). The method involves an enzyme‐linked immunosorbent assay in which the oxidatively damaged guanine species present in the sample compete with an 8‐hydroxy‐deoxyguanosine‐acetylcholinesterase conjugate for the binding sites of a monoclonal antibody, leading to an inverse correlation between the absorbance detected using spectrophotometer and the amount of oxidized guanine species present in the sample. The reaction product was read at 412 nm.

### 2.5 Determination of Cytokines

Interleukines (IL) 1β, 6, 8, and 10 were quantified in plasma using the commercial kit ProcartaPlex™ Human High Sensitivity Simplex (Invitrogen, item code MAN0019074). The method involves the utilization of beads coupled to antibodies to which the analytes bind to, followed by the addition of a detection antibody linked to streptavidin, a molecule that binds to biotin conjugated to a fluorophore. The analysis was performed using the equipment *Luminex xMAP*.

### 2.6 Determination of Nitrite/Nitrate

Nitrosative damage was evaluated in plasma samples using the commercial kit *Nitrate/Nitrite Colorimetric Assay* (Cayman Chemical, Code 780001). The method involves the use of nitrate reductase to convert the nitrate present in the sample to nitrite, followed by a Griess reaction that converts the nitrite into an azo product. The absorbance was read at 540 nm using spectrophotometer.

### 2.7 Determination of Cathepsin D

This biomarker was assessed in plasma samples using the commercial kit Human Cathepsin D ELISA (Cayman Chemical, code EH74RB). The method involves an enzyme‐linked immunosorbent assay in which the cathepsin D present in the sample is captured by a capture antibody. The subsequent steps involve the formation of a streptavidin‐biotin conjugate and the addition of a substrate that leads to the formation of a chromogenic product, proportional to the concentration of analyte present in the sample. The absorbance was read at 450 nm.

### 2.8 Statistical Analyses

Statistical analysis was performed using the GraphPad Prism Software (GraphPad Software Inc, San Diego, CA, USA—version 8.4.3). Means were analyzed by unpaired *t*‐test for all determinations. The *p* values above 0.05 were considered not statistically significant.

## 3 Results

Organic acid analysis indicated the presence of dicarboxylic, glutamic, methylmalonic, fumaric, ethylmalaric, adipic, and suberic acids in five out of 12 patients, with only two individuals exhibiting more than two of these acids. The clinical characteristics, metabolic status, and treatment of the patients included in the study are summarized in Table 1. The patient undergoing diagnostic evaluation showed a more severe profile, characterized by elevated levels of latic, 3‐hydroxy‐butiric, 2‐methyl‐3‐hydroxy‐butiric, 3‐hydroxy‐isovaleric, adipic, suberic, 3‐hydroxy‐sebacic, 3‐hydroxy‐dodecadienoic and 3‐hydroxy‐dihidro‐sebacic acids, indicating a metabolically decompensated state. The biomarker values obtained for this illustrative case are summarized in Table 2.

**Table 1:** Clinical characteristics, metabolic status, and treatment of FAOD patients included in the study.

Disorder	Age	Metabolic status at sample collection	Symptoms and signs at diagnosis	Additional treatment (besides dietary management and l‐car)
MADD	1 month	Stable	Enlarged kidneys, hypertrophic cardiomopathy, high anion gap metabolic acidosis, acute encephalopathy, seizures, dysmorphic features, growth restriction	NI
MADD	1 month	Stable	Hypoglycemia, high anion gap metabolic acidosis	NI
MADD	4 years	Stable	Seizures, hypoglycemia	Riboflavin (200 mg/day)
MADD	13 years	Stable	NI	Riboflavin
MADD	47 years	Stable	NI	NI
LCHADD	2 months	Stable	NI	Riboflavin
LCHADD	9 months	Stable	Frontotemporal atrophy, hypotony, dismorphic features, psychomotor developmental delay	NI
LCHADD	1 year	Stable	NI	NI
LCHADD	1 year	Decompensated	Hepatomegaly, hypoglycemia, hypotony, convulsions and delay in neuropsychomotor development and growth	NI
VLCADD	2 years	Stable	No signs of hepatic or muscular impairment. Adequate Antropometry and neuropsychomotor development	NI
VLCADD	6 years	Stable	NI	NI
VLCADD	11 years	Stable	NI	NI
MCADD	47 years	Stable	NI	NI

Abbreviation: NI, not informed.

**Table 2:** Oxidative stress and inflammatory biomarkers in the untreated patient at diagnosis (illustrative case).

Parameter	Plasmatic concentration
Nitrite/Nitrate	70.04 μM
DNA damage	19492.8 pg/mL
8‐Isoprostanes	34877.1 pg/mL
Cathepsin D	0.2646 ug/mL
IL‐1β	0.2495 pg/mL
IL‐6	64.023 pg/mL
IL‐8	73 pg/mL
IL‐10	3.231 pg/mL

Two patients undergoing treatment presented a slight reduction in free carnitine levels, whereas the recently diagnosed patient exhibited a marked decrease. Among the treated patients, only three did not present acylcarnitines, such as 3‐hydroxyhecadecanoil‐carnitine (C16‐OH), 3‐OH‐hexadecenoil‐carnitine (C16:1‐OH), and 3‐hydroxy‐octadecanoil‐carnitine (C18‐OH), which were found in the patient at the moment of diagnosis.

DNA/RNA damage was determined by the measuring oxidized guanine species in plasma samples (Figure 1), in which the mean concentrations did not differ significantly between the FAODs patients under treatment and the control group (9839.4 ± 1541.8 pg/mL, *n* = 11; 10999.7 ± 2751.8 pg/mL, *n* = 10, respectively). The patient at the moment of diagnosis presented 19,492.8 pg/mL, considerably increased in relation to the subjects in the previously described groups.

**Figure 1:**
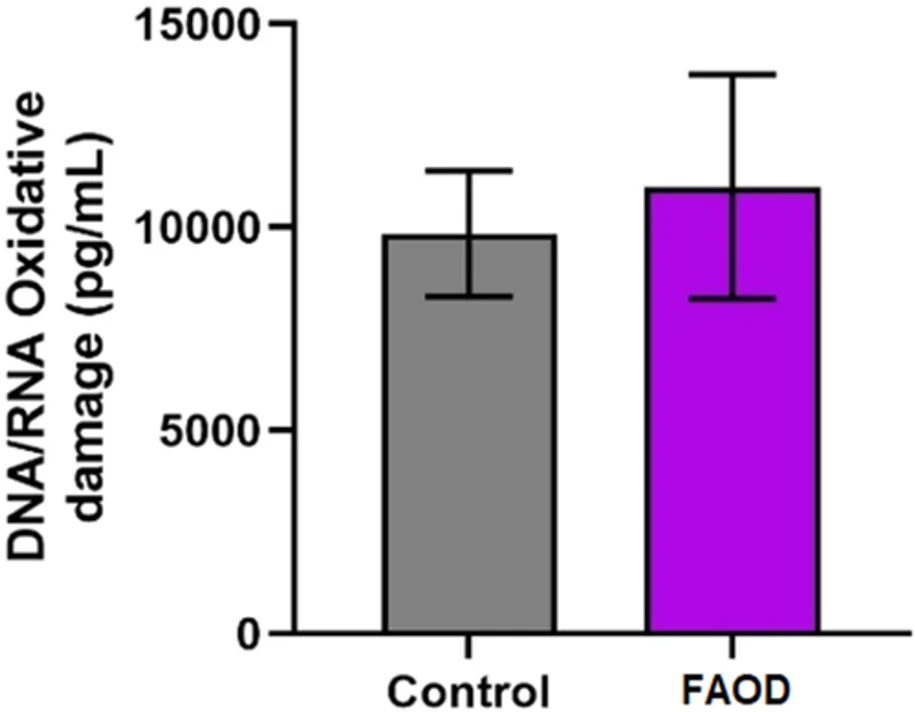
Plasma levels of oxidized guanine species in the control group (Control, *n* = 11, 9839.4 ± 1541.8 pg/mL) and patients under treatment for FAOD (FAOD, *n* = 12, 10,998.7 ± 2751.8 pg/mL). Results are expressed as mean ± SD. For the analysis, an unpaired *t*‐test was used. *p* < 0.05 was considered significant.

For nitrate/nitrite levels, which are indicative of oxidative stress, no significant differences were observed in the mean concentrations in plasma samples compared with the control group (50.50 ± 22.10 µM, *n* = 16), patients under treatment (48.89 ± 14.85 µM, *n* = 12) and the patient at the moment of diagnosis (70.04 µM). Meanwhile, patient at the moment of diagnosis presented an increased in plasma isoprostanes levels (34,877.1 pg/mL) in relation to the control group. However, the results showed no difference between the treated individuals and the control group (2634.23 ± 1658.60 pg/mL, *n* = 11; 3127.5 ± 1891.61 pg/mL, *n* = 11, respectively; Figure 2).

**Figure 2:**
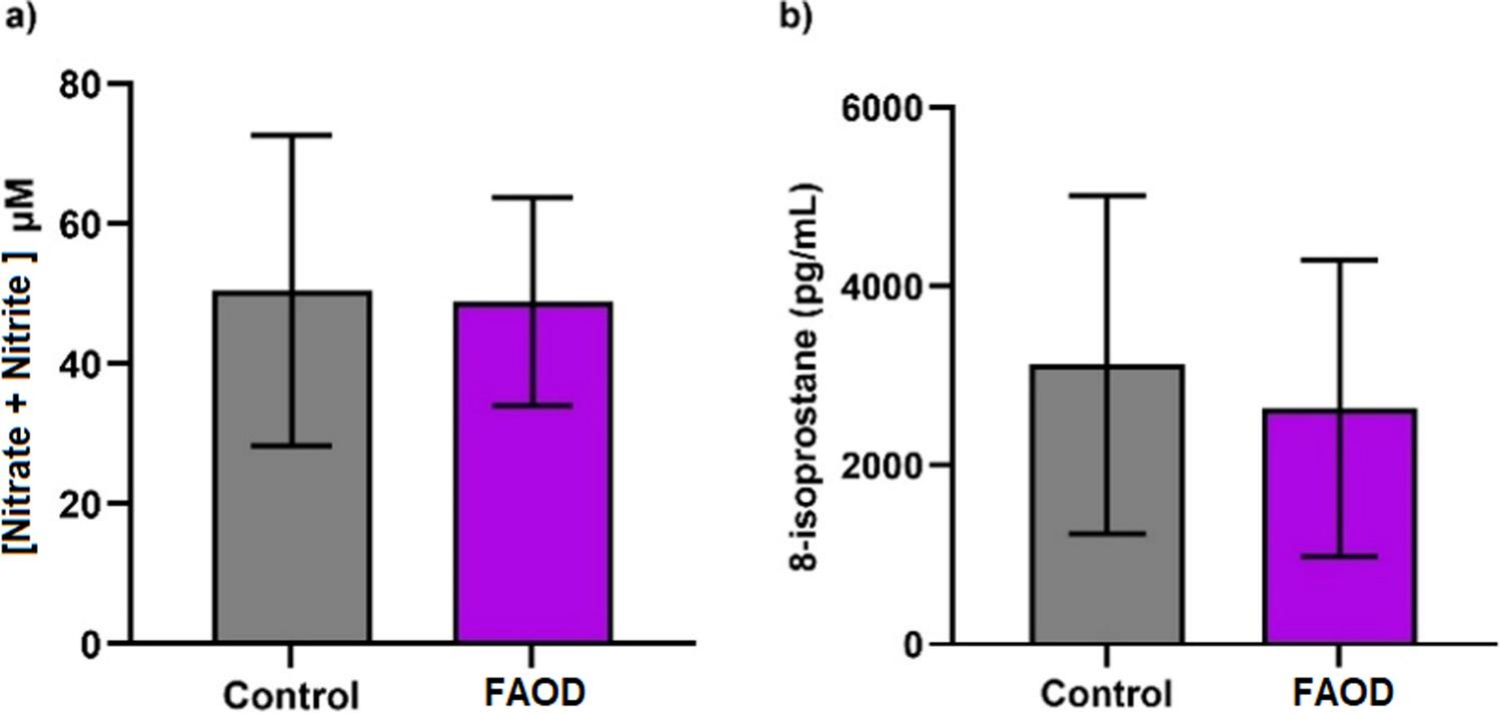
Plasma levels of nitrite and nitrates (a) and isoprostanes (b) in the control group (*n* = 16, 50.50 ± 22.10 µM and *n* = 9, 3127.5 ± 1891.61 pg/mL, respectively) and patients under treatment for FAODs (*n* = 12, 48.89 ± 14.85 µM and *n* = 11, 2634.2 ± 1658.60 pg/mL, respectively). Results are expressed as mean ± SD. For the analysis, an unpaired *t*‐test was used. *p* < 0.05 was considered significant.

The determination of IL‐6, a pro‐inflammatory cytokine, showed a statistically significant difference between the control group (0.125 ± 0.049 pg/mL, *n* = 13) and treated patients (0.318 ± 0.29 pg/mL, *n* = 9) in plasma samples, as shown in Figure 3a. No significant difference was found between treated patients and control group in concentrations of IL‐10 (0.100 ± 0.093 pg/mL, *n* = 9; 0.073 ± 0.058 pg/mL, *n* = 13, respectively; Figure 3b), IL‐1β (0.054 ± 0.040 pg/mL, *n* = 11; 0.027 ± 0.011 pg/mL, *n* = 16, respectively; Figure 4a) and, IL‐8 (0.320 ± 0.26 pg/mL, *n* = 11; 0.245 ± 0.26 pg/mL, *n* = 16, respectively; Figure 4b). The patient at the moment of diagnosis displayed a very different scenario, with increased levels in both IL‐6 and IL‐10 in relation to the groups (64.023 pg/mL and 3.231 pg/mL, respectively).

**Figure 3:**
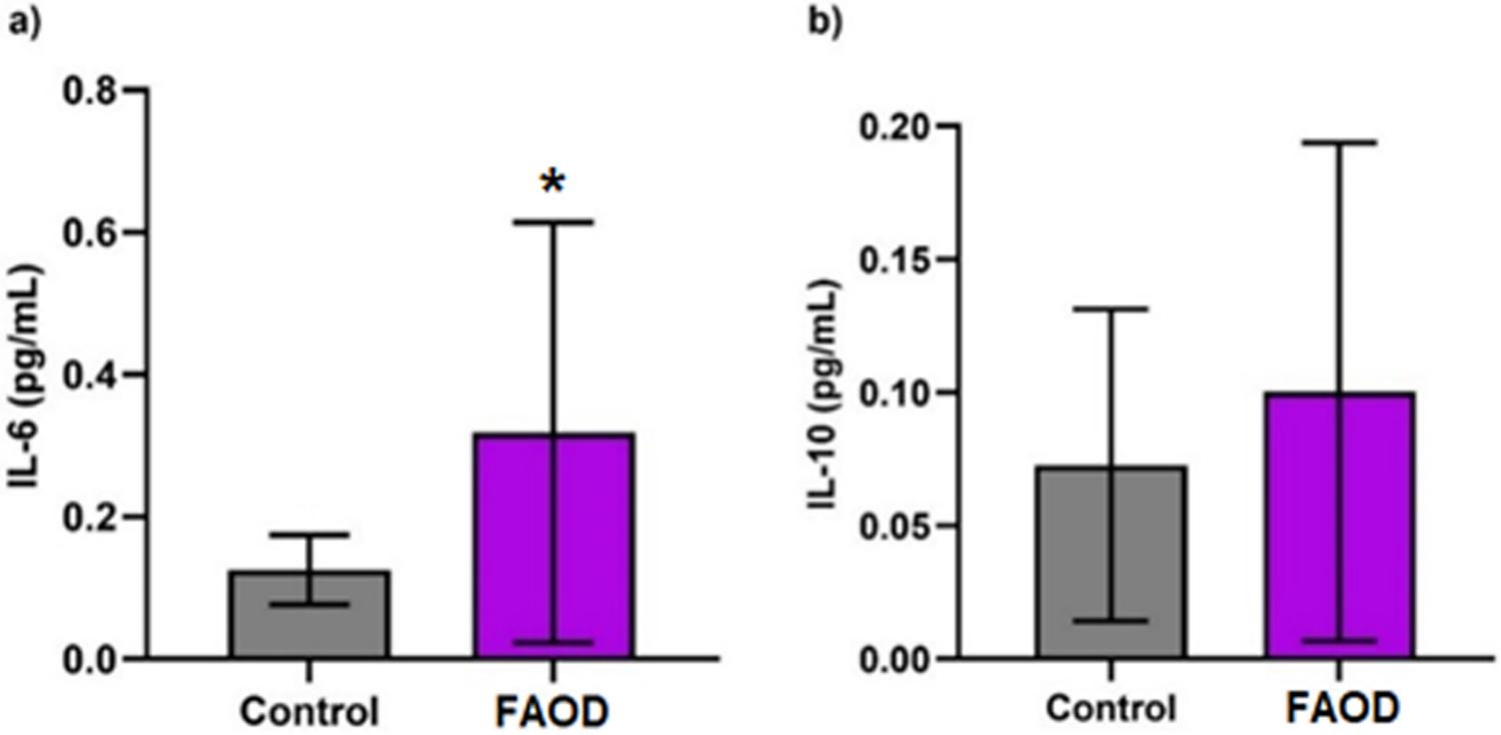
Plasma levels of IL‐6 (a) and IL‐10 (b) in the control group (*n* = 13, 0.125 ± 0.049 pg/mL and *n* = 16, 0.073 ± 0.058 pg/mL, respectively) and patients under treatment for FAOD (*n* = 9, 0.318 ± 0.29 pg/mL and *n* = 12, 0.100 ± 0.093 pg/mL, respectively). Results are expressed as mean ± SD. For the analysis, an unpaired *t*‐test was used. “*” Indicates statistical significance (*p* < 0.05).

**Figure 4:**
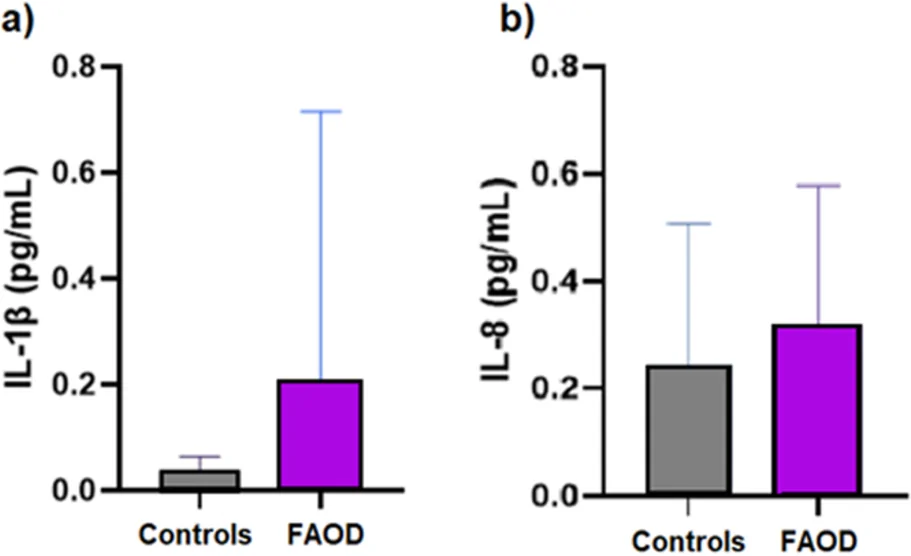
Plasma levels of IL‐1β (a) and IL‐8 (b) in the control group, *n* = 16 (0.027 ± 0.011 pg/mL and 0.246 ± 0.26 pg/mL) and patients under treatment for FAOD, *n* = 11 (0.054 ± 0.040 pg/mL and 0.320 ± 0.26 pg/mL). Results are expressed as mean ± SD. For the analysis, an unpaired *t*‐test was used. *p* < 0.05 was considered significant.

No significant difference were observed in Cathepsin D levels between treated patients and control group in plasma samples (0.189 ± 0.168 µg/mL, *n* = 15; 0.202 ± 0.078 µg/mL, *n* = 9; respectively; Figure 5). Likewise, the patient at the moment of diagnosis present a similar level of this marker (0.265 µg/mL), when descriptively compared to the other groups.

**Figure 5:**
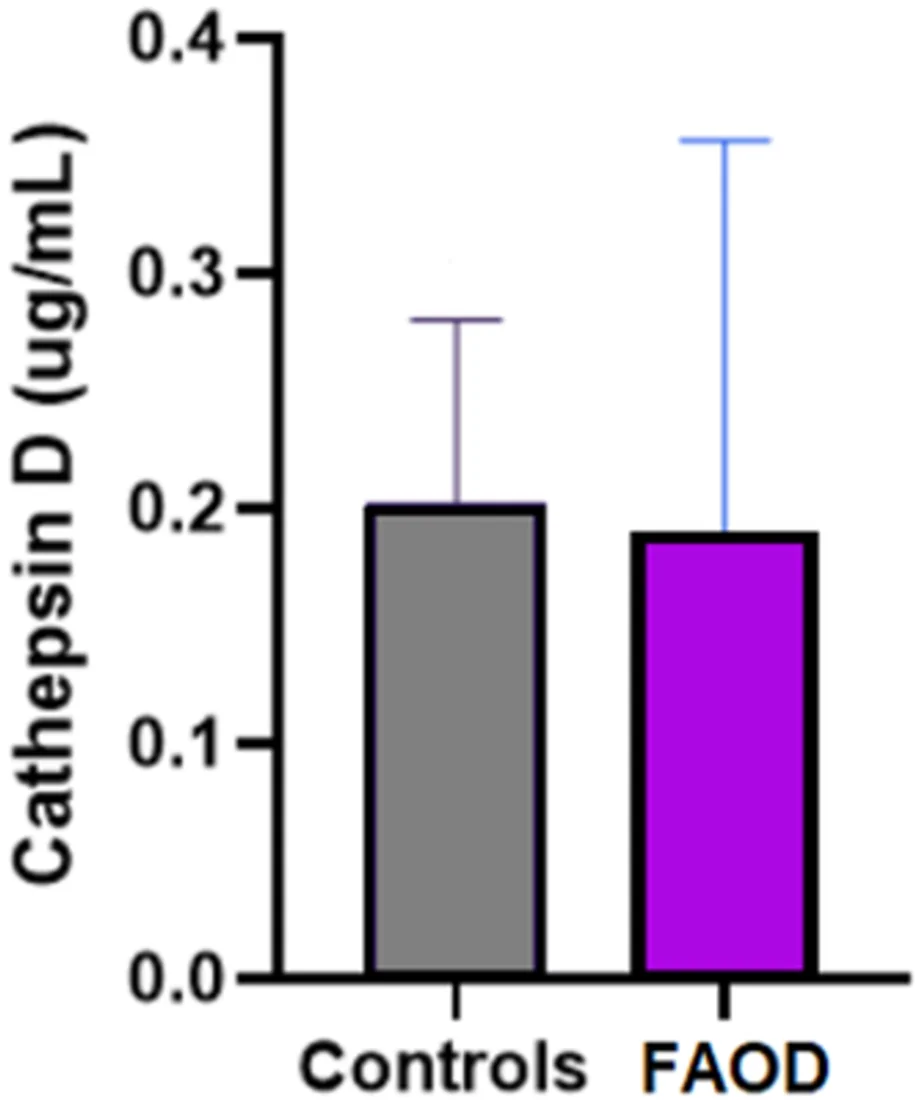
Plasma levels of Cathepsin D in the control group (*n* = 15, 0.202 ± 0.078 µg/mL) and patients under treatment for FAOD (*n* = 9, 0.189 ± 0.168 µg/mL). Results are expressed as mean ± SD. For the analysis, an unpaired *t*‐test was used. *p* < 0.05 was considered significant.

## 4 Discussion

Multiple conditions may contribute to the development of MD, which is strongly related to lipotoxicity, oxidative stress, and inflammatory processes [18, 21]. The accumulation of lipid metabolites and impairment of oxidative phosphorylation, as observed in FAODs, favor uncoupling and the production of ROS, leading to the oxidation of biomolecules essential for adequate mitochondrial function [8]. Among the reactive species formed, perhydroxyl radicals tend to initiate the peroxidation of polyunsaturated fatty acids, resulting in the formation of 8‐isoprostanes [22]. The relationship between lipid peroxidation and alterations in membrane function and permeability, as well as increased protein carbonylation and nucleic acid damage has been extensively described in the literature [23]. A previous study from our group reported elevated urinary levels of 8‐isoprostanes and oxidized guanine species in FAOD patients, specially LCHADD, during metabolic decompensation (at diagnosis moment) when compared to the control group, highlighting the role of oxidative stress in the pathophysiology of FAOD [14].

Our results indicate that dietary treatment may decrease oxidized guanosine species and 8‐isoprostanes in treated patients, when compared to the descriptive case of the patient at the moment of diagnosis, resulting in values comparable to those of the control group. These findings suggest a beneficial effect of this treatment on ROS generation and lipid peroxidation in plasma samples.

ROS are also known to contribute to the release of nitrogen reactive species (RNS), with nitric oxide (NO) being known as an important initiator, especially in the presence of O_2_^−^ [24]. Endothelial nitric oxide synthase (eNOS) directly impacts the production of RNS, while its activity is modulated by peroxynitrite, the most predominant and cytotoxic RNS [24, 25]. RNS have been shown to promote lipid peroxidation, inactivate mitochondrial proteins, and damage DNA and RNA [25]. Therefore, the occurrence of nitrosative stress in patients with RNS was an expected outcome.

However, in the present study, nitrite and nitrate levels did not differ significantly between patients and controls in plasma samples. Our previous study [14] reported similar findings, as only decompensated LCHADD patients presented increased nitrite and nitrate levels compared to control group. Although this may be due to the limited number of individuals evaluated, it could also reflect the impact of treatment on RNS generation. Given that only one untreated patient was included as an illustrative case, further investigations are needed in order to elucidate the role and the clinical relevance of nitrate and nitrite in FAODs.

Lipid metabolism is directly related to immune response, as lipid‐derived signaling modulate inflammation and macrophage function [26]. Under oxidative stress, the peroxidation of polyunsaturated fatty acids can also trigger the activation of nuclear factor kappa B (NF‐κB) and inflammasomes, leading to the release of pro‐inflammatory cytokines [27, 28]. Consequently, there appears to be a strong association between fatty acid oxidation defects in FAODs and inflammation.

Among the markers assessed, IL‐1β, IL‐8, and IL‐10 levels were comparable among groups and decreased when compared to the patient at the moment of diagnosis. IL‐1β is a pro‐inflammatory cytokine [29]. Although fatty acid oxidation contributes to IL‐1β secretion, this upregulation is also known to depend on ROS, which, as previously discussed, were not increased compared to the control group [30]. This finding may indicate that the treatment is associated with reduced inflammation in patients. On the other hand, IL‐10 has anti‐inflammatory effects, helping to restrict inflammation to avoid unnecessary cellular damage [31]. It is possible that, in metabolically decompensated patients, IL‐10 levels are increased as a compensatory mechanism, and after a period of treatment, inflammation is reduced and its concentration returns to basal [32, 33, 34]. IL‐8 promotes neutrophil recruitment and activation, and our results may be explained similarly to those observed for IL‐1β [35]. Regarding these findings, it is worthy of note the study of McCoin et al. [36], in which LCHAD patients receiving dietary treatment and supplementation with l‐carnitine presented increased levels of IL‐8 and decreased levels of IL‐10 when compared to healthy controls. The observed difference may be attributable to the pooling of patients with different FAODs in our study, which may mask the specificities of the blood cytokine pattern of LCHAD patients.

Treated patients showed increased levels of IL‐6. This interleukin is biologically implicated in the enhancement of lipolysis and β‐oxidation, while also increasing the insulin‐mediated glucose uptake by cells [37]. In this context, we suggest that the elevated IL‐6 levels in patients with FAODs may reflect a compensatory mechanism, in which impaired mitochondrial fatty acid oxidation triggers the release of this cytokine in order to reduce fatty acid accumulation. However, it may also contribute to the hypoketotic hypoglycaemia commonly found in these patients [38]. This could also be related to the lipotoxicity mechanisms previously mentioned, since it was observed that the patient at the moment of diagnosis presented substantially increased levels of IL‐6. Alternatively, IL‐6 elevation may reflect nonspecific factors such as residual inflammation, metabolic stress, or other physiological conditions. Therefore, the biological significance of isolated IL‐6 elevation in FAOD patients remains unclear. Although statistical analysis was not feasible, it is likely that current therapeutic interventions may be associated with the decrease of inflammatory cytokines. Further studies are required to better elucidate the role of IL‐6 in FAODs.

Cathepsin D is a lysosomal protease involved in proteostasis and pro‐apoptotic signaling. Oxidative stress promotes protein misfolding and truncation, with decreased cathepsin D levels being associated with inadequate proteolysis and neurodegeneration [39]. Physiologically, lipid peroxidation alters biological membranes, contributing to the release of this enzyme and the activation of neuronal apoptosis. Considering the occurrence of cerebral manifestations in FAODs, such as encephalopathy, and the involvement of oxidative stress, altered levels of this biomarker were expected [8]. Our findings may be influenced by the limited number of individuals evaluated, and additional studies are required to determine possible association between treatment and cathepsin D levels in plasma samples.

It is important to highlight that the patient at the moment of diagnosis displayed abnormal levels of organic acids, as seen in Moraes et al. [14], reinforcing the relevance of descriptively comparing the oxidative and inflammatory parameters of this type of patient with those belonging to patients under treatment.

Overall, most treated patients presented free carnitine levels within the reference range due to supplementation, in contrast to the patient at diagnosis moment. Carnitine plays a key role in regulating acyl‐CoA/CoA ratio and, consequently, mitochondrial enzymatic activity. It also facilitates the transport of long‐chain fatty acids into the mitochondrial matrix, allowing β‐oxidation [40]. Moreover, l‐carnitine is also thought to mitigate oxidative stress through different mechanisms, such as the activation of antioxidant enzymes transcription, inhibition of enzymes involved in reactive species production, and direct scavenging of toxic molecules that promote oxidative stress, leading to the acylcarnitine in FAOD patients, as previously described [41]. Our results suggest that l‐car may be associated with, at least partially, account for the comparable levels observed across the evaluated parameters.

## 5 Conclusions

In conclusion, our findings suggest that treated patients with FAODs present biomarker profiles indicative of reduced oxidative stress and inflammation, with most parameters similar to those of controls. To the best of our knowledge, this is the first study to evaluate oxidative and inflammatory parameters in treated patients with FAODs. Considering specially the small number of biological samples, further studies with a larger number of patients and complementary methodological approaches are necessary to better elucidate the mechanisms of this group of inborn errors of metabolism.

## Ethics Statement

This study was approved by the Ethics Comitee of Clinical Hospital of Porto Alegre (21‐0424). The study was conducted in accordance with the ethical standards of this approval committee and principles of the Declaration of Helsinki.

## Consent

All participants of this study were informed about the aims and procedures of this work and informed their consent prior to their inclusion.

## Conflicts of Interest

The authors declare no conflicts of interest.

## Data Availability

The data that support the findings of this study are available on request from the corresponding author. The data are not publicly available due to privacy or ethical restrictions.
